# Epilepsy Disease and Nose-to-Brain Delivery of Polymeric Nanoparticles: An Overview

**DOI:** 10.3390/pharmaceutics11030118

**Published:** 2019-03-13

**Authors:** Teresa Musumeci, Angela Bonaccorso, Giovanni Puglisi

**Affiliations:** Department of Drug Sciences, University of Catania; V.le Andrea Doria, 6, 95125 Catania, Italy; abonaccorso@unict.it (A.B.); puglisig@unict.it (G.P.)

**Keywords:** poly-lactide-co-glycolide, nanocarrier, intranasal, epilepsy, brain, pharmaceutical nanotechnology, anti-epiletic drug, nose to brain

## Abstract

Epilepsy is the fourth most common global neurological problem, which can be considered a spectrum disorder because of its various causes, seizure types, its ability to vary in severity and the impact from person to person, as well as its range of co-existing conditions. The approaches to drug therapy of epilepsy are directed at the control of symptoms by chronic administration of antiepileptic drugs (AEDs). These AEDs are administered orally or intravenously but alternative routes of administration are needed to overcome some important limits. Intranasal (IN) administration represents an attractive route because it is possible to reach the brain bypassing the blood brain barrier while the drug avoids first-pass metabolism. It is possible to obtain an increase in patient compliance for the easy and non-invasive route of administration. This route, however, has some drawbacks such as mucociliary clearance and the small volume that can be administered, in fact, only drugs that are efficacious at low doses can be considered. The drug also needs excellent aqueous solubility or must be able to be formulated using solubilizing agents. The use of nanomedicine formulations able to encapsulate active molecules represents a good strategy to overcome several limitations of this route and of conventional drugs. The aim of this review is to discuss the innovative application of nanomedicine for epilepsy treatment using nose-to-brain delivery with particular attention focused on polymeric nanoparticles to load drugs.

## 1. Introduction

Epilepsy is the fourth most common global neurological problem after migraine, stroke, and Alzheimer’s disease as reported by the World Health Organization (WHO) in its report “Epilepsy” in April 2018. It is possible to consider a person as epileptic when two or more unprovoked seizures occur [1]. After diagnosis, the etiology of epilepsy is often defined, but there are some cases where it is difficult to determine the causes. Treatment depends on the type of seizures considering other factors that could interfere such as the patient’s age, the co-administration of other drugs, and the side effects that appear in the patient. Epilepsy is not a single disorder but a spectrum of diseases that include different forms of comorbidity (depression, anxiety, learning disabilities, attention-deficit hyperactivity disorder, intellectual disability, and autism) [2]. To date, there are no therapies that have been shown to cure epilepsy. Thus, pharmacological treatment is directed at the control of symptoms by the chronic administration of anti-epileptic drugs (AEDs). Epileptic patients can receive treatment in emergency or during acute or chronic therapy. In the first case, it is desirable to have an immediate effect of the drug in the brain while in the second case there is a reduction of side effects and an increase of patient compliance. Different routes of administration can be considered for AEDs. The oral route is the most used for chronic treatment but cannot be considered, as the patient, during an epileptic attack, can suffer from vomiting or nausea. Rectal, buccal and parental routes represent valid alternatives to oral administration but each is characterized by several limits. The intranasal (IN) route represents an interesting solution for either acute or chronic treatment. It is an alternative to parenteral administration to obtain a rapid delivery of the drugs to the Central Nervous Systems (CNS) because it allows drugs to flow directly to the brain. IN administration could involve different pathways: the trigeminal and olfactory nerve and/or the systemic pathway. Each of these could contribute to the therapeutic effect, because the passage of the drug through the blood brain barrier (BBB) is also possible from the systemic pathway. However, this route reduces the effective dosage expected by other administration routes [3]. In 1989, William Frey II established the basis to consider IN administration as a strategy to bypass the BBB.

The administration of AEDs by the IN route is a new area of investigation as demonstrated by the benzodiazepine nasal sprays that are under clinical trials for the treatment of seizures. Various researchers have been comparing the effects obtained after the administration of AEDs by the conventional and the IN route. Preliminary results are very encouraging and promising. With the aim of achieving direct access into the brain, innovative devices have been developed and marketed (Optinose^®^, Bi-Directional™ technology), to channel the drug to the olfactory region in the upper site of the nose [4,5]. To achieve this goal it is relevant to obtain prevalent direct access to the brain. It is also well-known that IN administration involves different pathways, as reported by Illum et al. [6] In 2011 Xinfeng Liu wrote “Why do we need IN delivery?” today, the answer cannot be the same as when he posed the question [7]. On the one hand, it is true that there is an increased need to find safe, easy, and efficient methods to deliver drugs into the CNS, but, on the other hand, a great gap still exists between preclinical and clinical studies in this field. 

The IN treatment of some neurological disorders has given negative results during several clinical trials. These data should be interpreted in several ways: 1. drug inefficiency; 2. drugs unable to reach the brain following IN delivery; 3. the number of patients treated during the trial which is usually very small and not sufficiently sensitive. In some cases all these factors occurred, in other cases the negative results were due to an incorrect trial plan [7]. This should not discourage researchers to continue investigating this strategy [8]. The increasing number of published papers in this field allows us to declare that nose to brain delivery could really represent a valid strategy to treat epilepsy syndrome. Accordingly, the aim of this review is to show the state-of-the-art of drug delivery systems (DDS) for epilepsy treatment. Since a very recent review was published on the specific topic lipidic nanosystems and intranasal delivery of AEDs, we have excluded from our manuscript discussion on these nanosystems. In our review we focus on polymeric nanosystems without reviewing lipid nanoparticulate delivery strategies [9]. In fact, over the last decades, several IN formulations have been developed for this disorder. Commercial products have been investigated and clinical studies (phase I, II and III) are ongoing and in recruitment process/phase, as reported by Kapoor et al., [10]. Currently, there is a lively debate in the literature about the involved mechanisms in the transport of the drug from the nose to the brain. Generally, the number of research articles concerning nose to brain delivery (N2B) and epilepsy is limited, the published papers deal with brain disorders principally including Alzheimer or pain and only a few studies treat the delivery of AEDs. This is one of the reasons that the application of IN administration to epilepsy represents an innovative area to explore [11]. The reduced number of studies concerning N2B is also due to the drawbacks associated with this route. Indeed, IN administration offers several advantages such as easy drug delivery, however, it shows different limits, as reported below, due the anatomic structure of the site and the intrinsic physico-chemical properties of the molecules that have to be delivered. In order to overcome such limits, a key role can be played by the formulations themselves (e.g., excipients and/or structured carriers). The use of surfactants and or penetration enhancers represents a common way to increase drug solubility [12]. These strategies sometimes are insufficient to overcome the intrinsic limits associated with IN administration.

Moreover, despite the promising new AEDs available and the possibility to use IN administration as an alternative way to deliver drugs to the brain, the efficacy of epileptic treatment has not been achieved and seizures remain uncontrolled. The optimization of therapy could be obtained using new formulations able to reduce the interactions and severe effects due to conventional treatment. Innovative DDS could be used to promote prolonged/controlled release profiles of the drug for the treatment of patients during chronic therapy. This strategy could be particularly useful for pediatric patients. Prolonged and controlled release systems could reduce the number of drug administrations; increase the protection from seizures, and decrease side effects associated with conventional drugs. Thus, the use of traditional drugs loaded in innovative nano-carriers could overcome these disadvantages and improve the treatment of neurodegenerative diseases. In fact, nanoparticles (NPs) could protect drugs from rapid degradation and increase their residence time at the local site of administration. Furthermore NPs could improve drug uptake into cells from nose to brain through the anterograde way along axons of the olfactory or trigeminal nerves [12,13,14,15]. Another pathway that needs to be considered is crossing the olfactory mucosa, which plays an important role for the passage of the released molecules or NPs per se. These delivery carriers made from polymers, mainly polyesters and/or chitosan, or lipids, were designed as conventional or engineered systems to promote the transport of the loaded molecules across the neural pathway. The synergic effect of nanotechnology and N2B delivery could give new hope to patients affected by epileptic syndromes.

## 2. Epilepsy Pathophysiology

Epilepsy is a chronic CNS disorder in which nerve cell activity in the brain becomes disrupted, causing recurrent seizures or/and periods of strange behavior, sensations, and sometimes loss of consciousness with the highest incidence in both males and females, and can develop at any age. A first epileptic seizure occurs in 300,000 people each year; between 75,000 and 100,000 of these are children younger than five years of age who are experiencing febrile seizures. The incidence is highest in those younger than age two and those older than age 65, males are slightly more at risk than females. Economic conditions and origin of population influence the onset (African-American and socially disadvantaged populations are affected at a higher rate than Caucasians) [1]. Symptoms caused by epilepsy were described in ancient Babylon. For this reason it is considered one of the first brain diseases reported, the etymology of the word derived from the Greek that means “attack” [1,2]. Epileptic seizures arise from an excessively synchronous and sustained discharge of a group of neurons. Anything that disturbs the normal pattern of neuronal activity from illness to brain damage to abnormal brain development can lead to seizures. Indeed, epilepsy may have different causes, such as genetic factors, abnormality in brain wiring, an imbalance of nerve signaling in the brain, infection, traumatic brain injury, oxygen deprivation, stroke, brain tumors, and metabolic derangements but for about half of those with this condition no specific causative factors are found [16]. As a consequence, epilepsy can be considered a spectrum disorder because of its several causes, seizure types, its ability to vary in severity and the impact from person to person, and its range of co-existing conditions [17]. The single feature that unites all epileptic syndromes is a persistent increase of neuronal excitability. Generally, a person is not considered to have epilepsy until he/she has had two or more unprovoked seizures separated by at least 24 h. Seizures can be divided into three major groups as classified in the International Classification of Epileptic Seizures 2017: generalized onset, focal onset, which may become secondarily generalized, and unclassified seizures [18]. Generalized seizures represent approximately 30% of cases and are a result of abnormal neuronal activity that rapidly emerges on both hemispheres of the brain simultaneously. These seizures may cause loss of consciousness, falls, or massive muscle contractions. The many kinds of generalized seizures include: absence seizures, tonic seizures, clonic seizures, myoclonic seizures, atonic seizures, tonic-clonic seizures, and secondary generalized seizures [1]. Focal seizures originate and affect a limited area of one hemisphere of the brain and occur in approximately 60% of cases. Different areas of the brain (the frontal, temporal, parietal, and occipital lobes) are responsible for controlling all of our movements, body functions, feelings, and reactions. Therefore, focal seizures can cause many different symptoms. Partial seizures are split into two main categories; simple partial seizures and complex partial seizures. In simple partial seizures, a small part of one of the lobes may be affected and the person remains conscious but may experience motor, sensory, or psychic feelings. Instead, a complex partial seizure affects a larger part of the hemisphere than a simple partial seizure and the person may lose or have an alteration of consciousness, which can produce a dreamlike experience. Some people with focal seizures may experience an aura which is an unusual sensation that warns of an impending seizure [19]. The group of “unknown onset seizures”, as the name suggests, refers to all those cases when the nature of seizure onset is known with less than 80% confidence by the clinician. An unknown onset seizure could be later reclassified as focal or generalized as new information becomes available [18]. The seizure classification is not a classification of the epilepsies. An epilepsy classification is a multilevel classification which takes into account different information such as seizure type, epilepsy type, epilepsy syndrome, etiology, and comorbidities as reported and detailed by Scheffer et al. [20]. Although different epileptic syndromes differ pathophysiologically, ictogenesis-related mechanisms are often shared. It is now generally accepted that ictogenesis mainly results from neuronal membrane hyper-excitability. Both neurotransmitter systems and ion channels play a crucial role in neuronal excitability. In Engelborghs work, a deep study on the pathophysiology of epilepsy forms can be found, readers could refer to this review for further details [21]. Taking into account the extremely delicate condition, a prompt and accurate diagnosis with appropriate medical management will optimize the situation.

## 3. Diagnosis and Antiepileptic Drug Treatment of Epilepsy 

Over the last decades, advances have been made to the diagnostic approach of epilepsy. The main means to diagnose epilepsy is the medical history of the patient, taking into consideration information such as type of seizure and patient condition just before their onset. Physical examination, especially of the nervous system and analysis of blood and other body fluids, should be performed. Furthermore, a complete blood count, blood chemistry profiles, liver and thyroid function tests, an electroencephalogram, and a brain study, should be also included in the diagnosis program [1]. Innovative survey tools have been added over recent decades with the use of neuroimaging. These techniques include magnetic resonance tomography (MRI), positron emission tomography (PET), and single photon emission computed tomography (SPECT) while each has contributed to the diagnosis of pathological areas of the brain, such as tumors, cortical/subcortical dysgeneses, inflammation, strokes, vascular dysplasia, and post-traumatic insult. In addition, new imaging techniques in the diagnosis of epilepsy include functional MRI (fMRI), clinical proton MR spectroscopy, and magneto-encephalography (MEG) [22]. At this time, there are no therapies that have been shown to prevent epilepsy. In the absence of a specific etiological understanding, approaches to drug therapy of epilepsy must necessarily be directed at the control of symptoms by chronic administration of AEDs. Anti-convulsants are the main treatment option and consist of a group of drugs that are highly susceptible to drug–drug and drug–food interactions [23]; however, not all medications work for all types of epilepsy or for every individual [24]. The appropriate use of AEDs requires a deep understanding of their clinical pharmacology. Three major classes of mechanisms are recognized for anticonvulsant activity: modulation of ion channels, augmentation of inhibitory neurotransmission, and modulation of excitatory neurotransmission. The ion channels affected include the sodium and calcium channels. Augmentation in inhibitory neurotransmission includes increasing CNS concentrations of gamma-aminobutyric acid (GABA), whereas efforts to decrease excitatory neurotransmission are primarily focused on decreasing (or antagonizing) glutamate and aspartate neurotransmission [1]. Anticonvulsant drugs are grouped into three generations, categorized by the year in which they were developed and released. The first generation, entering the market from 1857 to 1958, includes potassium bromide, phenobarbital (PB), and a variety of drugs that were derived mainly by the modification of the barbiturate structure, including phenytoin (PHT), primidone (PRM), trimethadione, and ethosuximide (ESM). The second generation AEDs was introduced between 1960 and 1975 and include carbamazepine (CBZ), valproate (VPA), and the benzodiazepines, which differed chemically from the barbiturates. The third generation AEDs started in the 1980s with the development of progabide, vigabatrin (VGB), and tiagabine (TGB), drugs designed to target selectively a mechanism that was thought to be critical for the occurrence of epileptic seizures. Among new AEDs, it is worth mentioning oxcarbazepine (OXC), which is a keto analogue of CBZ and is a potent anticonvulsant used alone or in combination with other agents in the therapy of partial seizures. In particular, OXC is a second generation AED used for the treatment of partial seizures as a monotherapy or as an adjunctive therapy in adults and children aged 4–16 years [1]. OXC is sometimes used to treat acute mania in adults, as well as bipolar disorder and neuropathic pain. The mechanism of action for OXC is not completely understood but its activity is exerted through its active metabolite, in fact, OXC is rapidly reduced to 10,11-dihydro-10-hydroxy-carbazepine. This drug exerts different effects such as blockade of voltage-sensitive sodium channels, stabilization of hyperexcited neural membranes, inhibition of neural firing, and reduction of synaptic impulse propagation [25]. Similar to CBZ and OXC there is a new AED named eslicarbazepine acetate, a prodrug. Since 2009, eslicarbazepine acetate has been approved in Europe for adjunctive therapy and FDA approval arrived some years late. The prodrug is rapidly and extensively metabolized to eslicarbazepine (*S*-10-monohydroxy-oxcarbazepine) through hydrolysis during first-pass metabolism and its mechanism of action is related to the blockade of fast-acting, voltage-gated sodium channels responsible for neuronal signal propagation [26]. Despite these encouraging and welcome advantages that new AEDs have brought for the management of epilepsy, there is growing concern that the efficacy of drug treatment of epilepsy has not substantially improved with the introduction of new AEDs. However, seizures remain uncontrolled in at least 30% of all epilepsies despite adequate AED therapy [24,25,26,27,28]. Moreover, awareness of pharmacokinetic properties, side effects, indications, dosage forms, drug interactions (as mentioned above), and the AED metabolic pathway, as well as inducer or inhibitory effects on the liver, can help in the optimization of AED therapy. Pharmacokinetic interactions are a common complicating factor in AED selection. Interactions can occur during pharmacokinetic processes. Because of interactions, caution should be used when AEDs are added to a drug regimen [29]. Careful and rigorous diagnosis and classification of seizure and syndrome type is critical to select the most suitable pharmacotherapy. Moreover, patient characteristics such as age, comorbid conditions, ability to comply with the prescribed regimen, and presence or absence of insurance coverage can also influence the choice of AEDs. Poly-therapy should be avoided if possible, but it is inevitable in approximately 30–50% of patients who fail to respond to single-drug therapy. It is important to underline that special groups of patients require particular attention and management such as children, the elderly, pregnant women, and people with mental and physical disabilities because they are vulnerable and their treatment is more demanding [17,18,19]. Moreover, epilepsy therapy is commonly continued for many years and is often life-long, thus chronic adverse effects must be considered. Based on this evidence, novel agents are necessary because about a third of patients continue to be pharmaco-resistant. Different hypotheses have been discussed on refractory epilepsy such as the pharmacokinetic hypothesis, the neural network hypothesis, the intrinsic severity hypothesis, the gene variant hypothesis, the target hypothesis, and finally the transporter hypothesis [30]. This demonstrates a continued need for developing new formulations with the aim of creating new concepts and original ideas to effectively prevent epilepsy or its progression. Recent ongoing clinical trials of epilepsy can be found in an interesting review published by Kaur et al., in which the current developmental status of new drugs for treatment of epilepsy and a brief description about antiepileptic drugs that are targeting different mechanisms were reported [31]. IN delivery of drugs on antiepileptic treatment or innovative formulations could overcome some of these drawbacks.

## 4. Antiepileptic Drugs: Limits during Treatment 

The treatment of acute and chronic seizure disorders requires administration of antiepileptic drugs by multiple routes. For chronic treatment of epilepsy, antiepileptic drugs are commonly administered orally [32]. Parenteral routes are used when the oral route is unavailable or a rapid clinical response is required. Some drugs can also be administered by the buccal, sublingual or IN routes, especially for out-of-hospital treatment [33]. Oral formulations include solutions, suspensions, tablets, capsules, and extended release products. Orally administered drugs absorbed by the gastrointestinal (GI) tract reach the liver, prior to entering the systemic circulation, where first-pass metabolism of a drug can occur resulting in decreased bioavailability. Thus, drugs having low bioavailability due to extensive first-pass metabolism or low GI absorption cannot be administered orally because of the possibility of high inter-subject variability in the relationship between dose and concentration. Buccal and sublingual routes bypassing gastric and hepatic first-pass metabolism are ideal for lipid-soluble drugs that are highly metabolized in the GI tract or liver [34]. Furthermore, part of the drug can be swallowed and thus the bioavailability can be incomplete and the time to peak concentration delayed. The major disadvantage of mucosal administration is that patient cooperation is required, which may not be feasible during an epileptic seizure attack. Maintaining therapeutic concentrations of the AED is necessary for optimal seizure control. When the oral route is not available, such as in the presence of intractable nausea and vomiting, drug allergies, and when intravenous administration is not possible, alternative routes are needed. Emergency treatment, such as status epilepticus, requires intravenous formulations [35]. In the treatment of out-of-hospital acute seizures and in situations when the parenteral route is not available, caregivers can use buccal, IN, and rectal routes safely. The rectal route can be considered a valid alternative to the oral route for the pediatric population because these dosage forms are to be neither swallowed nor need to be taste-masked. Rectal forms can also be administered in an emergency to unconscious children [36]. As reported in the study of Anderson consensus documents from the Task Force on Status Epilepticus of the International League against Epilepsy (ILAE) and others recommend rectal diazepam, buccal midazolam, or IN midazolam for the out of-hospital treatment of early seizures. Instead, in the United States, the FDA has only approved one rectal AED formulation, Diastat^®^, for home use [32]. As mentioned, there are a number of novel antiepileptic compounds that are under various stages of drug development and their current developmental status for treatment of epilepsy can be found in Kaur’s study [31]. These new drugs under clinical study are expected to provide better treatment options for epilepsy in terms of improved pharmacokinetics, safety, and efficacy. Brivaracetam was approved in 2016 by the European Medicine Agency (EMA) and then by the U.S. FDA. It was approved under the trade name Briviact (developed and marketed by UCB) as an add-on treatment to other medications to treat partial onset seizures in patients age 16 years and older with epilepsy. Crossing the BBB remains the main obstacle in the development of drugs for brain diseases despite decades of research. The BBB is formed of endothelial cells lining the brain microvessels, thus, drugs acting in the brain, including AEDs, are generally lipophilic, to be able to cross the brain endothelium via lipid membranes. However, such lipophilic drugs are potential substrates for efflux carriers of the BBB, such as P glycoprotein (Pgp), located on the endothelial luminal membrane. It is estimated that up to 50% of drug candidates may be substrates for Pgp that is, an ATP-dependent drug efflux pump and a member of the ATP-binding cassette (ABC) transporter superfamily. ABC drug efflux transporters are important elements of the BBB. In fact, these transporters restrict the permeability of a large number of toxins as well as therapeutic agents. Among these, Pgp, Breast Cancer Resistance Protein (BCRP), Multidrug resistance-protein (MRP1) and (MRP2) are the most studied [37] ABC efflux transporters present neuroprotective action, however, they could also restrict the entry of many drugs and contribute to CNS pharmaco-resistance. Alterations in ABC efflux transporter expression and activity occur in several neurological diseases. In fact, pharmaco-resistance to AEDs for epilepsy treatment is also associated with the overexpression of these transporters by extruding AEDs from their target site [38]. An interesting approach proposed to decrease the activity of ABC efflux transporters in neurological diseases including epilepsy is the use of direct inhibitors in order to promote a transient inhibition of ABC efflux transporters to prevent pharmaco-resistance due to overexpression of such transporters [39]. Several inhibitors of Pgp, BCRP- and MRPs have been developed, however, most of these inhibitors lack specificity and inhibit several ABC efflux transporters while they exhibit side effects at high concentrations. Moreover, the expression and activity of ABC efflux transporters in neurological diseases can be affected by different factors (progression of disease, alterations in target sites and modifications in the expression of other proteins) thus, to target ABC efflux transporters in the treatment of neurological diseases it is necessary to understand the specific mechanisms that regulate individual transporters. Finally, treatment with AEDs could also induce pharmaco-resistance in epilepsy, however, data are still controversial [37]. An interesting study by Grewal et al., [38] showed that the regulation of ABC transporters can be affected by the oxidative stress generated during epilepsy or to the use of AEDs. Oxidative stress is an emerging event in the pathophysiology of epilepsy and AEDs can modulate pro-oxidant/antioxidant balance. The overexpression of ABC efflux transporters plays a critical role in limiting access to AEDs, the fact that oxidative stress generated during the course of the disease or due to AED treatment alters transporter expression at the blood-brain barrier level is a crucial point to understand the drug resistance phenomenon. The alteration may result in either overexpression of transporters contributing to efflux of AEDs or downregulation culminating in increased cellular toxicity due to reduced efflux of oxidative molecules [38]. To date, the transporter hypothesis is still controversial, and further research is needed to determine the clinical relevance of efflux transporter overexpression at the BBB [30].

This complicates pharmacological treatment and underlines the need for new therapeutic strategies [40]. As stated above, the route of administration is essential to achieve a successful treatment. IN administration may represent a good choice for brain delivery, it also bypasses gastric and hepatic first-pass metabolism even if this route of administration can be considered for drugs that are highly concentrated as the dose must be administered in a 100–200-μL spray or solution. The drug also needs excellent aqueous solubility or must be able to be formulated using solubilizing agents [41]. Nasal therapy could be an effective remedy to treat seizure emergencies. Spraying a benzodiazepine into the nose of someone who is having a seizure may potentially be one of the most sensible solutions. Over the last few years, there has been great interest in developing an FDA-approved benzodiazepine nasal spray. Currently, there are benzodiazepine nasal spray clinical trials underway for people with seizures. Over the next few years, there is an expectation that the FDA will approve a benzodiazepine nasal spray and make it available to people with epilepsy. There is a significant need for alternative and new formulations with the aim of decreasing the frequency of dosing of drugs with rapid elimination and of improving convenience and patients’ compliance especially in infants and children as well as in the elderly.

## 5. Intranasal Administration to Reach the Brain

Commonly, the IN route has been used to administer topically acting molecules to treat local diseases, such as allergies and nasal congestion. Over the last decades, IN administration has gained great attention in research and has been investigated extensively with regard to its feasibility to serve as a direct drug transport route to the CNS [4]. The interest in the IN route for therapeutic purposes arises from the particular anatomical, physiological, and histological characteristics of the nasal cavity [42]. In fact, the olfactory neuroepithelium located inside the nasal cavity is the only area of the body in direct contact with both the CNS and the external environment, which opens it up for therapeutic treatments. The therapeutic compound can be directly delivered to the brain by IN administration, as suggested and patented (Neurologic agents for nasal administration to the brain) by William H. Frey II (Frey patent). Frey is the Director of the Alzheimer’s Research Center, he developed an “Intranasal Insulin” treatment that was shown in multiple human clinical trials to improve memory and functioning in Alzheimer’s patients (http://www.alzheimersinfo.org/AlzheimerInfo/ourstory). The efficacy of the IN route has been demonstrated in several studies [43,44,45,46,47,48]. Deferoxamine is an approved generic drug that binds iron, Professor Frey and his collaborators discovered that IN deferoxamine bypasses the BBB to treat and prevent brain damage in animal models of Parkinson’s, Alzheimer’s, stroke, and other neurological disorders. An IN deferoxamine safety clinical trial was conducted in humans and showed no significant side effects [48]. Moreover, Professor Frey and his collaborators discovered that therapeutic cells, such as adult stem cells, immune cells and genetically-engineered cells, can be delivered to the brain using the IN delivery method. IN administration of stem cells bypassing the BBB, traveling along the olfactory neural pathway. Once in the brain, adult stem cells were shown to target the damaged areas of the brain to treat the disease. Stem cell treatment by the IN route has been efficiently applied in animal models of neonatal cerebral ischemia, neonatal brain damage and subarachnoid hemorrhage, and stroke. IN adult neural stem cells have also been shown to improve an animal model of multiple sclerosis [49]. In summary, this delivery and treatment method can facilitate the development of adult stem cell, immune cell, and genetically-engineered cell therapies for Alzheimer’s, Parkinson’s, stroke, multiple sclerosis, amyotrophic lateral sclerosis, progressive supranuclear palsy, Huntington’s, neonatal ischemia, brain tumors, traumatic brain injury, and spinal cord injury. Therefore, IN administration can be defined as a non-invasive method for bypassing the BBB and rapidly delivering and targeting therapeutic agents to the brain along the olfactory and trigeminal neural pathways. This alternative method to deliver drugs to the brain has gained great interest of both pharmaceutical companies and neuroscientists. Today the idea of Professor Frey is considered a valid alternative with the aim of overcoming the BBB and reaching brain areas. As mentioned above, IN administration avoids the GI tract and hepatic metabolism, bypasses the BBB enhancing drug bioavailability and allowing a lower therapeutic drug dose and fewer systemic side effects [50]. Moreover, it also offers several practical advantages and only some limitations as shown in Table 1. Hence, it seems to be an encouraging route for the treatment of acute and chronic conditions requiring considerable drug exposure such as epilepsy, which require a life-long treatment or a rapid action. 

To date, the exact mechanisms of nose to brain delivery have not been entirely understood. Pathways involving nerves connecting the nasal passages to the brain and spinal cord are important as well as pathways involving the vasculature, cerebrospinal fluid, and lymphatic system. Thus, it seems that a combination of these pathways should be responsible, one may predominate depending on the properties of the drug, the characteristics of the formulations, and the delivery device used. Pathways and mechanisms involved in nose-to-brain transport have already been extensively evaluated and can be found in the reviews of Illum, Kozlovskaya, Pires or Pardeshi and Belgamwar and in cited papers [3,42,46,51,52]. We report a brief summary of the most supported hypotheses. The delivery of substances from the nose to the CNS may occur via olfactory neuroepithelium and may involve paracellular, transcellular, and/or neuronal transport. The paracellular pathway refers to the transfer of substances across an epithelium through tight junctions between sustentacular cells or the clefts between sustentacular cells and olfactory neurons. This route is slow and passive, it is responsible for the transport of hydrophilic drugs, and it shows rate dependency on the molecular weight of a molecule [46]. The transcellular process is responsible for the transport of lipophilic drugs that show a rate dependency on their lipophilicity. This pathway involves transport across the sustentacular cells, most likely by receptor-mediated endocytosis, fluid phase endocytosis, or by passive diffusion. It is rapidly mediated and at a high rate [53]. 

The neuronal pathway consists of the transport of the drug into the neuronal cell by endocytosis or pinocytosis mechanisms and transported by intracellular axonal transport to the olfactory bulb [3,46]. The contribution made by the trigeminal pathway to IN delivery to the CNS has been recognized, especially to caudal brain regions and the spinal cord. Thorne and colleagues demonstrated the involvement of the trigeminal pathway studying the delivery of an insulin-like growth factor-I (125I-IGF-I) directly into the CNS following IN administration [54]. The authors found high levels of radioactivity in the trigeminal nerve branches, trigeminal ganglion, pons, and olfactory bulbs, consistent with delivery along both trigeminal and olfactory nerves. The trigeminal nerve innervates the respiratory and olfactory epithelium of the nasal cavity, enters the CNS in the pons, and represents another important pathway connecting nasal cavity to the CNS. Interestingly, a small portion of the trigeminal nerve also terminates in the olfactory bulbs. The trigeminal nerve pathway also plays a key role in the distribution of intra-nasally administered drugs to brain areas distant from the olfactory bulbs [42]. The ophthalmic and maxillary branches of the trigeminal nerve are important for nose-to-brain drug delivery as neurons from these branches pass directly through the nasal mucosa. The trigeminal nerve enters the brain from the respiratory epithelium of the nasal passages at two sites: through the anterior lacerated foramen near the pons and through the cribriform plate near the olfactory bulb, creating entry points into both caudal and rostral brain areas following IN administration [55]. Considering that part of the trigeminal neural pathway enters the brain through the cribriform plate alongside the olfactory pathway, it is difficult to distinguish whether intra-nasally administered drugs reach the olfactory bulb and other rostral brain areas via the olfactory or trigeminal pathways or if both are involved [46,56]. In addition to these direct pathways, transport may also occur via blood vasculature, lymphatics, and cerebrospinal fluid present in the nasal mucosa tissue. The nasal mucosa is highly vascularized, with a high density of blood vessels in the respiratory mucosa resulting in an ideal site for absorption into the blood. After the absorption into the systemic circulation, the drug has to cross the BBB to reach the CNS. However, many problems may arise with systemic delivery due to drug elimination via hepatic and renal mechanisms, and some other limiting factors such as the BBB, drug binding to plasma proteins, degradation by plasma proteases, and potential peripheral side effects [42].

We should consider that only drugs with specific properties can be administered intra-nasally to achieve this goal, i.e., lipophilicity, molecular weight (<20 KDa) and degree of ionization can increase drug transport into the CNS after IN administration. The active efflux transporter pumps at the apical membrane surface (Pgp) or enzymatic degradation in the olfactory epithelium could affect the transport of small molecular weight molecules. Moreover, the nasal route gives the opportunity to reduce the necessary effective dose, and considering the low volume of the nasal cavity, two requirements are necessary: the drug should be effective at low dosage and the drug solubility in water must be high enough to accommodate the necessary dose, necessitating highly concentrated nasal drug solutions [57]. In order to gain high bioavailability, the drug must be resistant to metabolizing enzymes in the nasal environment. In fact, a drug administered into the nasal cavity showed a metabolic capacity lower than in the liver or gastrointestinal (GI) tract. The part of the nasal cavity that is subjected to the highest metabolic capacity is the nasal mucosa thus nasally applied drugs might still be subjected to metabolisms in the nasal mucosa. Different enzymes have been recognized in human nasal tissue such as cytochrome P-450s (CYP) and epoxide hydroxylase, as well as UDP-glucuronyltransferase and glutathione trans-ferase. Metabolisms in the nasal cavity play important roles in the bioactivation of prodrugs, however, the impact of enzymatic degradation for nasal delivery of peptides is even more pronounced than for small molecules [58]. 

Finally, drug residence time, in contact with the mucosal membrane, is an important factor influencing drug absorption and has to be considered. 

As reported in scientific literature, there is evidence of the drug directly delivered to the brain after IN administration in appropriate conditions. Lipophilicity and the molecular weight of the drug can influence the possibility to reach the brain respecting the therapeutic dose, but some experimental evidence has shown that the amount of the therapeutic molecule in the CNS is normally less than 1% of the drug administered [59,60]. Westin and colleagues, in their study, showed that morphine can be transferred along the olfactory pathway to the CNS, in particular the authors found [^3^H]-morphine in the CNS surrounding the olfactory bulbs by autoradiography in rats within five minutes of IN administration [60]. However, no significant penetration of the radioactivity was detected in deeper brain areas. Below we provide a summary of the results of the studies carried out with AEDs administered through the IN route to reach the brain. 

### AEDs by IN Route: State-of-the-Art

AEDs may be administered intra-nasally as evidenced in several published papers. Different studies can be found in which the pharmacokinetics of anticonvulsants has been evaluated after IN administration. Serralheiro and colleagues, for example, performed an interesting study by using CBZ. The pharmacokinetics of CBZ after IN and intravenous (IV) administration to mice was evaluated and they investigated whether direct transport of the drug from nose to brain could be involved. The similar pharmacokinetic profiles obtained in all matrices following both administration routes indicate that, after IN delivery, CBZ quickly and extensively reaches the bloodstream, reaching the brain predominantly via systemic circulation. However, the uneven biodistribution of CBZ through the brain regions with higher concentrations in the olfactory bulb and frontal cortex following IN instillation, in comparison with the homogenous brain distribution pattern after IV injection, strongly suggests the involvement of direct transport of CBZ from nose to brain [61]. In the study of Kang et al., the efficacy of IN delivery of pentoxifylline, which is a potent antioxidant and modulator of a variety of transmitters, was investigated in epileptic seizures by analyzing the alteration of the mesodopaminergic system and hippocampus in status epilepticus rats induced by lithium-pilocarpine. For comparison purposes, the same observations were performed in status epilepticus rats that underwent intraperitoneal pentoxifylline treatment. The authors found that IN delivery of pentoxifylline to rats significantly suppressed the epileptic seizures induced by lithium-pilocarpine, ameliorated the deficits in visuospatial memory and in the mesodopaminergic system, and enhanced the transient activation of nuclear factor erythroid 2-related factor 2-(Nrf2-) in status epilepticus rats. Compared with intraperitoneal injection, IN pentoxifylline delivery completely restored the visuospatial memory and the activity of the mesodopaminergic system in status epilepticus rats. The findings obtained suggest that IN administration of pentoxifylline could effectively protect cells from oxidative damage in status epilepticus and may hopefully become a non-invasive, painless, and easily administered option for epileptic patients [62].

Several drugs have been successfully used to treat seizure emergencies. Benzodiazepines (BZDs) such as lorazepam (LZP), diazepam (DZP), and midazolam (MDZ) have been used thanks to their potency and rapid onset of action. BZDs can be administered intravenously, but this route requires hospitalization or the presence of skilled medical personnel. Moreover, this can result in a delay in treatment, which can induce brain damage [10]. As reported in the literature, IN midazolam is safe and effective for treatment of acute seizures in children, and quicker than intravenous diazepam to stop seizures. IN midazolam should be considered as a convenient anticonvulsant agent when intravenous access is not available [63]. Thanks to the advantages of the IN route, several nasal formulations for seizure emergency treatment are under clinical trials. The drugs under phase I, II, or III clinical trials are clonazepam, diazepam, and midazolam [10]. In order to enhance the amount of AEDs in the brain and consequently improve its pharmacological activity new strategies can be considered with the help of nanotechnology. Another study performed by Westin and colleagues showed that morphine can be transferred along the olfactory pathway to the CNS, in particular, the authors found [^3^H]-morphine in the CNS surrounding the olfactory bulbs by autoradiography in rats within five minutes of IN administration [60]. Another interesting result is shown by Veronesi et al. [64]. In this study, thyrotropin-releasing hormone (TRH) is intra-nasally administered to reduce total seizure ADD in rats. Different patients with untreatable epilepsies use TRH as an anticonvulsant drug with success, its effects in several animal seizure models was demonstrated. A wide application of this molecule is limited due to low bioavailability and difficulty in penetrating the BBB that reduces the duration of TRH action. However, no significant penetration of the radioactivity was detected in deeper brain areas. Despite some successful results, the design of the formulation represents a key step in overcoming the several challenges of nasal delivery: nasal degradation enzymes, limited nasal volume, mucociliary clearance, nasal blood flow, PgP efflux transporters, and physical factors. 

The optimization of nasal administration using drug delivery systems (DDS) may represent a strategy to increase the concentration of drug that reaches the brain after IN administration. DDS may improve drug properties for nasal delivery and can also modify the drug release profile, absorption, distribution, and elimination improving product efficacy and safety, as well as patient convenience and compliance reducing the number of the daily doses [10]. According to our records, Gao et al. was the first group to prove in 2007 that poly(ethylene glycol)-poly(lactic acid) NPs (PEG-PLA NPs) functionalized with wheat germ agglutinin (WGA) could deliver a neuropeptide against Alzheimer’s disease directly from the nose to the brain [65,66]. Since then, different nano-based drug delivery systems have been developed with the objective of improving the nose-to-brain delivery of a variety of peptide drugs [67,68]. 

## 6. The Device: A Key Tool to Achieve the Goal

One important condition for a molecule or a nanomedicine to reach the brain is to arrive in the upper portion of the nasal cavity. Conventional nasal spray devices cannot cover the entire olfactory region and their use has been limited primarily to treating nasal allergies. Innovative devices have been proposed and marketed by different companies for nose-to-brain delivery. Patient-friendly Exhalation Delivery Systems, proposed by Optinose, overcome the limits of traditional nasal sprays. It is possible to use the device for both powder and liquid formulations [4,5,69]. 

The first device for humans used for brain through nose is Vianase™ for IN insulin delivery. Vianase™ is an electronic atomizer device that is made up of a nebulizer attached to a vortex chamber. This structure supports the olfactory region deposition of the formulation and maximizes transport to the brain [69]. It is important to underline that the literature reports different definitions of the upper region, which are not readily comparable [70]. Therefore, it is difficult to establish the most efficacious devices.

## 7. “3N” Rule: Nasal Route, Nanomedicine, and Neurotherapy 

**Figure 1:**
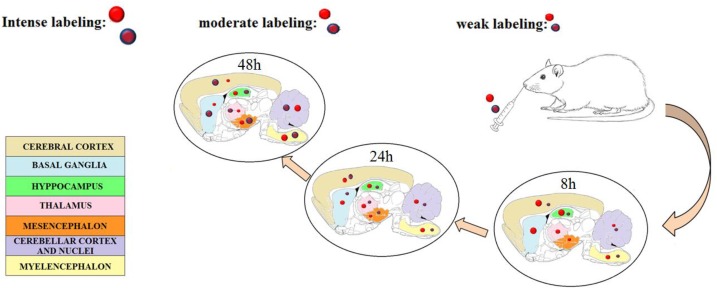
A scheme illustrating the localization of positive (red balls with blue ring) and negative (red balls) surface charged polymeric nanoparticles after IN administration in different brain regions (ex-vivo study in rats) at different times. The size of the balls indicates the intensity of fluorescence on the area by visual observation. [71] (By Bonaccorso et al., 2017) Reprinted with permission of Elsevier.

Over the last decades, the nasal route has represented a valid and alternative route of administration for molecules to reach the brain bypassing the BBB as above mentioned. To overcome some limits with the administration of the free drug the use of nanomedicine has been proposed. The combination of nasal route, nanomedicine, and neurotherapeutics is currently a relevant topic in research, these three nouns could become a single solution for brain targeting (Figure 1). Nanomedicine is a relevant field in the development of innovative formulations and it has received increasing attention by regulatory affairs. When nanomedicine is used, important questions emerge: Is the formulation a simple vehicle and the loaded drug released in a controlled manner in the site of administration? Are the molecule loaded NPs transported together into the brain? Both hypotheses are reported and data has demonstrated that in relation to the type of NPs and their properties one prevails over the other. Nanomedicine formulations are drug delivery systems with a size below 1000 nm obtained from different raw materials: phospholipids (liposomes), lipids (SLN, NLC), and polymers (nanocapsules, nanospheres, micelles). They could have different shapes, even if spherical is preferable for various applications, different sizes, and surface characteristics. Different types of nanosystems were investigated in pre-clinical studies, from screening of a suitable formulation to reach the brain intra-nasally to in vitro, ex vivo, and in vivo studies on the health or pathological model of animals. 

Considering nose to brain delivery nanomedicine, the literature has reported different raw materials used to obtain the colloidal carriers that are suitable to protect the drugs, control release, and prolong the residence time in the site of administration. Chitosan is one of the most studied thanks to its muco-adhesive and penetration enhancing properties [13,72]. Muco-adhesion is an important property that could have two effects: increased residence time for sustained release and/or retarded direct transport of NPs through axons [15,57,73]. Bonaccorso et al. reported that chitosan coated NPs are able to increase the residence time in the nose but retard the possibility of NPs reaching the brain. In fact, comparing the localization of two different types of fluorescent NPs in different sub-regions of the brain, in ex-vivo experiments in healthy rats, they showed fluorescence at 24 h in rostral regions for PLGA nanoparticles and at 48 h in caudal regions for chitosan coated PLGA NPs, as represented [71]. PEG chains are also able to increase residence time, thus another suitable material for nose to brain delivery could be different types of copolymers derivatized with PEG [74,75]. Lipid NPs obtained with tristearin in association with gliceryl monoolein were produced to load neuroactive drugs such as dimethylfumarate, retinyl palmitate, progesterone, and the endocannabinoid hydrolysis inhibitor URB597 [76]. Dimethyl fumarate was loaded in three different types of SLN for potential treatment of multiple sclerosis. Esposito et al. studied the biodistribution of polysorbate 80 treated SLN by fluorescent imaging after intraperitoneal or IN administration in mice [76]. Shah et al. studied rivastigmine loaded SLN optimized by design of experiment (DoE) using different lipids (Apifil, Compritol glycerylmonostearate, stearic acid). In this work the authors performed a histopathology study on nasal mucosa for potential IN use [77]. In the next section, we summarize the most important studies carried out with AED loaded drug delivery systems.

## 8. IN Delivery of AED Loaded Polymeric Nanoparticles

**Figure 2:**
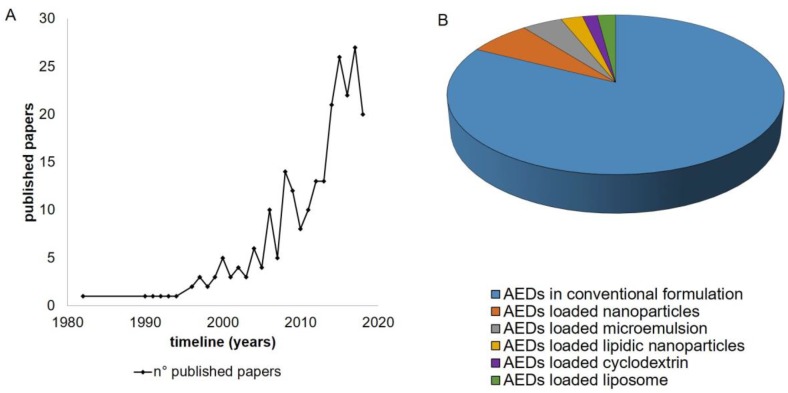
Publication trends in the field of antiepileptics and epilepsy (**A**), and the percentage of each type of nanomedicine with refined terms: nanoparticles, microemulsion, cyclodextrins, liposome, and solid lipid nanoparticles (SciFinder) (**B**). AEDs—antiepileptics’ drugs.

The investigation for this review was performed using SCIFINDER as the database considering the keywords: “IN and epilepsy” with the refining words (NPs, microemulsion, cyclodextrins, liposome, solid lipid NPs). Figure 2 shows the trends since 1982 considering the use of the IN route also as a diagnostic route or speculative route of investigation for epilepsy (A), over the last 20 years the field has attracted increasing interest by the scientific community. The percentage of published papers that deals with nanomedicine and epilepsy using the IN route is very few with respect to the total number found with selected keywords. For the discussion of the selected papers we pay particular attention to studies that deal with drug loaded polymeric nanocarriers and we invite the readers to examine the very recently published review regarding lipid-based nanosystems, such as solid lipid nanoparticles (SLN) and nanostructured lipid carriers (NLC), liposomes, nanoemulsions, and microemulsions [9]. As concerns cyclodextrins, we found two patents for formulations that claimed the potential use of complexes of the drug cyclodextrin for epilepsy. 

Nanomedicine is used to encapsulate AEDs to improve the therapeutic dose at the target site (brain) and reduce side effects due to the reduced bio-distribution on peripheral organs. A field of particular interest is using NPs for the delivery of AEDs via IN administration. NPs could provide an excellent platform for direct transport of drugs to the brain in relation to different characteristics. Physico-chemical properties of NPs influence their fate in the brain after IN administration as demonstrated by several authors. It is known that transport of NPs depends on size, shape, surface, and stability properties (the 4S rules) [78]. Among different formulations, NPs with a particle size below 300 nm are designed to load and deliver several drugs through the olfactory pathway to the brain. Several animal studies have shown that NPs delivered intra-nasally may enhance brain uptake, improve pharmacological activity or therapeutic efficacy, and reduce side effects of drugs compared to conventional DDSs. From the literature, it is evident that there is some difficulty to understand the exact mechanism of direct transport of NPs from the nose to the brain. In several studies, the authors carried out experiments without establishing which pathway was involved. In fact, it is possible to support two theories, one is that the drug is released from the nanocarrier in the nasal cavity and then transported to the brain and the other that drug-loaded nanomedicine is directly delivered via a neural connection. In a recent review, Tyler P. Crowe et al., treated the multiple factors that influence the transport of molecules to the brain after IN administration [79]. Another important factor to be considered is the potency of the drug, in fact, it is possible to speculate the use of only molecules that are highly concentrated (<20 mg per dose), as the dose must be administered in a 100–200 μL spray or solution. This shows the importance of high aqueous solubility, if this is not the case it has to be formulated using solubilizing agents. In the case of AEDs, examining benzodiazepines we know that they have limited aqueous solubility at pH 5–7. Encapsulation of the drug in a nanomedicine product can improve aqueous solubility. In this case the total mass of product (nanoparticle and drug) should be administered in the same volume, the drug should be potent to low concentration (<1–2 mg per dose) to reach the brain at a therapeutic concentration. In Table 2, we report the nanosystem approaches applied to deliver AEDs through the nose to reach the brain with particular attention to in vitro and in vivo studies. 

Nanomedicine combined with IN administration was studied to obtain two goals: a rapid delivery to the brain in the case of acute treatment or a prolonged release of the drug and reduced number of administrations in the case of chronic treatment. Vyes Tushar et al. prepared clonazepam microemulsions for IN administration to reach the brain. They studied the biodistribution of the drug in the brain using the radiolabeled form with 99mTc (technetium), a comparative evaluation was carried out on Swiss albino rats after IN and IV administrations. Their results showed that brain/blood uptake ratios indicated more effective targeting with IN administration and best targeting of the brain with an IN clonazepam loaded microemulsion at 30 min after IN or IV administrations. After 8 h from IN administration, the brain/blood ratio, at all sampling points, compared to IV administration was higher demonstrating a wide distribution of the molecules in the brain. [80] Veronesi Micheal et al. studied the opportunity to use TRH to significantly reduce several seizure parameters in an animal model, but the duration of protection lasted only up to one hour [64]. It is evident that sustained bioavailability is essential for a more durable therapeutic effect. Taking into account this limit, TRH was loaded into poly(lactic-acid) (PLA) NPs. The research team carried out the localization of dye NPs after IN administration [81]. They also studied the physiological effects examining protection against glutamate toxicity in vitro and the capability of suppressing a number of seizure characteristics in the rat kindling model [82]. PLA and poly-lactic acid-*co*-glycolide, (PLGA) are very biodegradable and biocompatible polymers, approved by the FDA for human use in 2004, they are among the most useful polymers to prepare polymeric NPs to load hydrophobic molecules. Their promising results encouraged further development of sustained-release anticonvulsant drug delivery in the form of NPs to the brain through the nasal route. Six years later, Sharma et al. selected diazepam to load into a PLGA matrix. Diazepam was selected based on the investigation of Ivaturi et al. that reported an alternative route to increase the bioavailability of this drug in 12 healthy volunteers. Ivaturi et al. compared IN diazepam and rectal diazepam and they found that diazepam showed a higher Cmax (181.8 ± 84 ng/mL) after intranasal administration compared to rectal gel (160.9 ± 109.4 ng/mL). The IN diazepam formulation can offer a feasible alternative to rectal administration of diazepam in the management of epilepsy seizures [83]. Sharma et al. carried out an ex-vivo study on sheep mucosa to evaluate drug release behavior of the drug from the matrix and cytotoxicity through the MTT test. The authors reported the biodistribution evaluation of the drug through Gamma Scintigraphy Imaging on Sprague-Dawley rats using diazepam radiolabeled ^99m^Tc. The study evidenced that ^99m^Tc-Diazepam-NPs had significantly higher brain uptake of the drug when compared to IN free diazepam and IV ^99m^Tc-Diazepam-NPs in healthy rats [84]. A similar study was carried out with lorazepam loaded PLGA NPs, where gamma scintigraphy images showed clear evidence of the high uptake of NPs in the brain after IN administration [85]. Pitavastatin was studied as an alternative or adjuvant therapy for epilepsy. For this reason Iqubal Ashifet et al. evaluated the antiepileptic properties of solid-lipid nanoparticles (SLNs) of pitavastatin (PTS) via the IN route, because the major limitation of statins, when myopathy is exerted, is dose related. The authors performed electroshock seizure (ICES) and maximal electroshock seizure (MES) tests to assess the antiepileptic activities of the nanosystem and they demonstrated that pitavastatin loaded SLNs were a valid alternative or adjuvant therapy for epilepsy treatment when the route of administration was the nose [86]. Encouraging results were obtained with lamotrigine loaded NLCs [87]. Among AED drugs, OXC is a promising molecule for epilepsy treatment. In spite of this, clinical use requires high oral doses that cause several side effects, for this reason OXC is an ideal molecule to be loaded into nanosystems. Moreover, experimental evidence has shown a higher reduction of the required dose to control seizures in rats when the free drug was administered intra-nasally. Musumeci et al. showed that IN administrations of OXC loaded PLGA NPs reduced the number of administrations to 1 in 24 h compared to the free drug, thus controlling seizures in rats. Translocation of dye loaded NPs was shown through Fluorescence Molecular Tomography, which also evidenced an accumulation of NPs in the brain after repeated IN administrations. These encouraging results confirmed the possibility of developing a novel non-invasive nose to brain delivery system of OXC for the treatment of epilepsy [88]. To evidence direct transport of molecules after IN administration it is relevant to calculate two important parameters: drug targeting efficiency (DTE%), which represents the time average partitioning ratio (Equation (1)), and direct nose-to-brain transport percentage (DTP%) (Equation (2)):

(1) $$DTE\%IN=\frac{(AUC_{brain}/AUC_{blood})IN}{\begin{array}{c} (AUC_{brain}/AUC_{blood)IV} \end{array}}\times 100$$

(2) $$DTP\%=\frac{\left(B_{IN}-B_{X}\right)}{B_{IN}}\times 100\;B_{X}=\frac{B_{IV}}{P_{IV}}\times P_{IN}$$

where B_IN_ is the AUC_max_ (brain) following IN administration, B_X_ is the brain AUC fraction contributed by systemic circulation through the BBB following IN administration, B_IV_ is the AUC_max_ (brain) following IV administration, P_IV_ is AUC_max_ (blood) following IV administration, and P_IN_ is AUC_max_ (blood) following IN instillation. These parameters were used by El-Zaafarany as proof of concept to evaluate brain targeting efficiency of OXC loaded nano-tryglycerid carrier defined emulsomal vesicles and, recently, OXC loaded emulsome-loaded poly(lactic acid-*co*-glycolic acid)–poly(ethylene glycol)–poly(lactic acid-*co*-glycolic acid) (PLGA–PEG–PLGA, triblock polymer) thermogel solutions [89,90]. In El-Zaafarany’s work, emulsomes were prepared as nanocarriers because of their particular morphology. Emulsomes showed a lipid core in both the solid and the liquid crystalline state at 25 °C, coated with a phospholipid bilayer. These nanocarriers combining the characteristics of lipid spheres (apolar core) and liposomes (hydrophilic surface) useful for lipophilic incorporation, encapsulation of emulsomes into thermoresponsive gel have been studied to improve the interesting results obtained in 2016. The gelification allowed the design of IN formulations with the ability to exhibit retention within the nasal cavity with constant exposure to mucocilliary clearance and enzymatic degradation. In particular, the long residence time in the plasma of drug loaded emulsomes compared to OXC solution could be due to the intrinsic lipophilic nature of the carrier. The potential partitioning of OXC loaded into or released from emulsome particles into the nasal epithelial cell membranes involved systemic circulation as a pathway to reach the brain. An interesting solution proposed to reduce nasal mucociliary clearance, one of the drawbacks for nasal drug delivery, is the use of a drug carrier with enclosed magnetic NPs. These nanosystems could be guided with the help of an external magnetic field to the target tissue. Superparamagnetic iron oxide NPs (SPION) are synthetic particles with a very small core (<100 nm in diameter) [91]. Kaur Sarabjit et al. bio-engineered NPs for encapsulation of TRH-analogues. They prepared biodegradable chitosan coated PLGA NPs to increase mucoadhesive properties. Quantum dots were used as fluorescent probes to evaluate the capability to reach the brain after IN administration. This methodology increases the mean diameter of prepared NPs because of the different sizes in nanometers of dots compared to drugs. The results showed the efficient uptake of NPs in the brain using the nose as the administration route [92].

Recently Asha Paul et al., developed an IN lamotrigine loaded in situ gel formulation. In this work, different polymeric solutions of gellan and xanthan gum were prepared to load lamotrigine. The optimized formulation was safe and nontoxic, on analyzing histopathological studies of nasal mucosa after permeation. Biodegradable polymeric solutions of gellan gum and xanthan gum increased the mucoadhesion and residence time and obtained an in situ gel drug delivery system with immediate release of lamotrigine to treat epilepsy. This formulation could minimize first-pass metabolism and side effects and reduce mucocillary clearance [93].

## 9. Conclusions

The diagnosis and therapy of epilepsy are very complex and the number of patients that suffer from this pathology are increasing. In fact, several causes are responsible for the outbreak of pathologies such as tumors or neurotherapies for neurodegenerative diseases. It is necessary not only to discover new drugs but also to re-formulate consolidated therapy to obtain a reduction of side effects, an increase of patient compliance, especially during chronic therapy, and to cross the BBB, which is a relevant challenge for CNS therapy. Currently, an emergent strategy to overcome the BBB is nose-to-brain delivery. IN delivery of AEDs has been proposed and discussed in this review. To overcome some of the limits shown with nasal administration, nanomedicine has been prepared, optimized and well characterized by several authors. Controlled and sustained release nanosystems are a valid solution to answer this necessity. The combination of nose and nanomedicine is the potential solution for neurotherapeutics to achieve the above mentioned aim (3N rule). The increasing number of in vivo studies published over the last few years on the IN delivery of AEDs indicates the growing interest in this route. Nanomedicine represents an interesting strategy to overcome some of the major limits, combined with the IN route of administration as investigated in preclinical studies. There are very few studies regarding polymeric nanosystems but still after examining these papers, we could establish that the use of nanocarriers could bypass the BBB and reach the brain. 

The major challenges are the evaluation of the pathway involved and the mechanism for arriving in the CNS. We hope that in a short time the efforts and successes obtained in preclinical studies can be translated into human clinical studies. This could represent a valid goal to control the pathology with a smart system for all people suffering epilepsy.

## Figures and Tables

**Table 1:** Advantages and disadvantages of Intranasal (IN) drug delivery.

Advantages	Disadvantages
Rapid, safe, non-invasive and convenient method	Rapid elimination of drug substances from nasal cavity due to mucocilliary clearance
Avoids drug degradation in the gastrointestinal (GI) tract, first-pass metabolism allowing enhanced bioavailability	Nasal congestion due to cold or allergic condition may interfere with this technique of drug delivery
Reduction of systemic side effects	Suitable for potent drugs since only a limited volume can be sprayed into the nasal cavity
Bioavailability for low molecular weight drugs	Frequent use of this route may lead to mucosal damage and/or irritation of nasal mucosa
Rapid drug absorption via highly vascularized mucosa	Mechanical loss of the dosage form could occur due to improper administration technique
Easy administration	Mechanisms of drug transport are still unclear

**Table 2:** Nanosystem approaches applied to the delivery of antiepileptic drugs (AEDs) through the nose to reach the brain with particular attention to in vitro and in vivo studies.

Drug	Raw Material	Type of Nanocarrier	In Vitro Study		Ex-Vivo/In Vivo Study	Animals	Authors	Year
			Method	Aim				
Clonazepam	-	Microemulsion	Trasmission electronic microscopy (TEM)	Uptake across the nasal mucosa	Scintigraphy	Rabbits/rats	Vyes Tushar et al., [80]	2006
TRH *	PLA	Nanoparticles (NPs)	Cell viability on fetal hippocampal neurons through Cell Proliferation Assay	Effect of TRH NPs on glutamate toxicity in cultured hippocampal neurons	Kindling development	Rats	Veronesi, Michael C et al., [81]	2009
			-		Immunostaining method to localize NPs into the brain	Rats	Kubek M. et al., [82]	2009
Lorazepam	PLGA	Nanoparticles	Cell viability on Vero cell line through MTT assay	Evaluation of the safety profile of optimized NPs	Gamma scintigraphy and biodistribution	Sprague-Dawley Rats	Sharma D. et al. [85]	2014
Carbamazepine	-	Microemulsion	-		Scintigraphy	Rats	Rashmin Bharatbhai Patel et al., [94]	2014
Diazepam	PLGA	Nanoparticles	Cytotoxicity on Vero cell lines through Cell Proliferation Assay	Evaluation of potential toxicity of DZP as free drug or loaded in PLGA NP	Ex-vivo drug release; gamma scintigraphy and biodistribution	Sprague-Dawley Rats	Sharma D. et al. [84]	2015
Pitavastatin	-	SLN	-		Electroshock seizure (ICES) and maximal electroshock seizure (MES)	Mice	Iqubal Ashif et al., [86]	2015
Lamotrigine	-	NLC	-		In vivo efficacy and scintigraphic study	Wistar rat model	Alam, Tausif et al., [87]	2015
Oxcarbazepine	PC	Nano-tryglycerid carrier	Cell viability on Calu-3-nasal cells using Cell Proliferation Assay	Effect of different concentrations of OXC, free and drug loaded TO17-Tw	Pharmacokinetic studies	Rats	Ghada M. El-Zaafarany et al. [89]	2016
Oxcarbazepine	PLGA-PEG-PLGA and PC	Emulsome loaded nanoparticles	Mucoadhesion study on nasal mucosa using a texture analyser	Demonstrate the ability to exhibit retention within the nasal cavity with the constant exposure to mucocilliary clearance and enzymatic degradation.	Pharmacokinetics, residence time and histopathology examination of nasal tissues	Rats	Ghada M. El-Zaafarany et al. [90]	2018
Oxcarbazepine	PLGA	Nanoparticles	-		Tomography, pharmacokinetics and behavioral study	Epilepsy model in rodents	Musumeci et al. [88]	2018
Clonazepam		SPIO-lipid nanoparticles	-		Anticonvulsant action	Swiss albino mice	Abbas A. et al. [91]	2018
TRH * analogues	PLGA	Nanoparticles	Cells viability by MTT assay against HaCaT cell line.	Determine toxicity of nanoparticles in in-vitro system	Localization of nanoparticles	Male Sprague Dawley	Kaur, Sarabjit et al., [92]	2018
Lamotrigine	Gellan gum and Xanthan gum	In situ gel	-		Ex-vivo permeation studies; Histopathological study of nasal mucosa after permeation	Bovine nasal mucosal tissue	Asha Paul et al., [93]	2017

* TRH—Thyrotropin-releasing hormone.
